# Laterally Excited Bulk Acoustic Wave Resonators with Rotated Electrodes Using X-Cut LiNbO_3_ Thin-Film Substrates

**DOI:** 10.3390/s25061740

**Published:** 2025-03-11

**Authors:** Jieyu Liu, Wenjuan Liu, Zhiwei Wen, Min Zeng, Yao Cai, Chengliang Sun

**Affiliations:** 1 The Institute of Technological Sciences, Wuhan University, Wuhan 430072, China; jieyuuu@whu.edu.cn (J.L.); zwei_wen@whu.edu.cn (Z.W.); zengmm@whu.edu.cn (M.Z.); caiyao999@whu.edu.cn (Y.C.); 2 Hubei Key Laboratory of Electronic Manufacturing and Packaging Integration, Wuhan University, Wuhan 430072, China; 3 Wuhan Institute of Quantum Technology, Wuhan 430072, China

**Keywords:** X-cut LiNbO_3_ thin film, angle rotation, IDT

## Abstract

With the development of piezoelectric-on-insulator (POI) substrates, X-cut LiNbO_3_ thin-film resonators with interdigital transducers are widely investigated due to their adjustable resonant frequency (*f_s_*) and effective electromechanical coupling coefficient ($K_{eff}^{2}$). This paper presents an in-depth study of simulations and measurements of laterally excited bulk acoustic wave resonators based on an X-cut LiNbO_3_/SiO_2_/Si substrate and a LiNbO_3_ thin film to analyze the effects of electrode angle rotation (θ) on the modes, *f_s_*, and $K_{eff}^{2}$. The rotated θ leads to different electric field directions, causing mode changes, where the resonators without cavities are longitudinal leaky SAWs (LLSAWs, θ = 0°) and zero-order shear horizontal SAWs (SH_0_-SAWs, θ = 90°) and the resonators with cavities are zero-order-symmetry (S_0_) lateral vibrating resonators (LVRs, θ = 0°) and SH_0_ plate wave resonators (PAW, θ = 90°). The resonators are fabricated based on a 400 nm X-cut LiNbO_3_ thin-film substrate, and the measured results are consistent with those from the simulation. The fabricated LLSAW and SH_0_-SAW without cavities show a $K_{eff}^{2}$ of 1.62% and 26.6% and a Bode-*Q_max_* of 1309 and 228, respectively. Meanwhile, an S_0_ LVR and an SH_0_-PAW with cavities present a $K_{eff}^{2}$ of 4.82% and 27.66% and a Bode-*Q_max_* of 3289 and 289, respectively. In addition, the TCF with a different rotated θ is also measured and analyzed. This paper systematically analyzes resonators on X-cut LiNbO_3_ thin-film substrates and provides potential strategies for multi-band and multi-bandwidth filters.

## 1. Introduction

With the rapid development of technology, innovations and breakthroughs in wireless communication technology are also constantly occurring. Fifth-generation (5G) mobile communication technology provides stronger support for future applications such as digitization, intelligence, and networking [1]. With the continuous development of communication, the division of communication frequency bands is becoming increasingly refined and complex; therefore, the requirements for the diversity and performance of radio-frequency (RF) components such as filters are also increasing. The demand for integrating multi-band and multi-bandwidth filters in limited space is increasing.

Lithium niobate (LiNbO_3_) piezoelectric materials have significant differences in piezoelectric properties due to their crystal properties, and materials with different tangential directions also exhibit significant variations in their piezoelectric properties [1]. Thanks to the development and widespread application of ion-slicing technology, LiNbO_3_ piezoelectric materials are gradually being applied in filters [2,3,4,5,6,7,8,9,10,11]. There are many studies on the LiNbO_3_ substrate with different tangential directions [12,13,14,15,16,17,18,19,20,21]. Resonators based on the Z-cut LiNbO_3_ thin film are commonly used at high frequencies and wide bandwidths due to their large piezoelectric coefficient e_16_. The laterally excited bulk acoustic wave resonator (XBAR) based on a Z-cut LiNbO_3_ substrate exhibits an excellent electromechanical coupling coefficient (*K*^2^) larger than 20% when operating at 5 GHz [22,23,24]. Other types of resonators based on LiNbO_3_ substrates have also been extensively studied. Fabricated film bulk acoustic resonators (FBARs) using a Y+36°-cut LiNbO_3_ substrate working at 4.7 GHz and with an effective electromechanical coupling coefficient ($K_{eff}^{2}$) of 25% and a quality factor at anti-resonance of 574 have been reported [6]. One study of resonators based on rotated Y-cut LiNbO_3_ on the *K*^2^ of the shear horizontal (SH) mode showed that the largest *K*^2^ is 15% [25].

In recent years, the resonators with LiNbO_3_ plates bonded on different substrates such as silicon carbide (SiC) have attracted great interest [26,27,28,29,30]. Generally, silicon (Si) substrates are also typically chosen as long as they are low cost and can support the confined propagation of surface acoustic waves (SAWs) in LiNbO_3_ due to the sharp contrast of material properties between Si and LiNbO_3_. A study on the SAWs on a 15°YX-LiNbO_3_/SiO_2_/Si substrate presented a high *K*^2^ ranging from 22.5% to 25.2% [31]. S_0_ mode laterally vibrating resonators (LVRs) on an X-cut LiNbO_3_ substrate operating between 100 MHz and 1 GHz have been widely investigated [32]. But there is still an urgent need for systematic research on resonators based on X-cut LiNbO_3_ thin-film substrates that meet the needs of wideband filtering.

In this paper, the performance of the resonators based on an X-cut LiNbO_3_ thin film based on an X-cut LiNbO_3_/SiO_2_/Si substrate and a LiNbO_3_ thin film are investigated. The mode, resonant frequencies, and the $K_{eff}^{2}$ of different modes corresponding to resonators with different angle rotations (θ) were carefully studied and analyzed. Further simulation analyses were conducted on the resonant main modes corresponding to resonators with different values of θ. The fabricated devices are systematically compared, and the simulation results are summarized. In addition, in this article, we also study the TCF of resonators under different values of θ.

## 2. Modeling and Simulation

### 2.1. Device Modeling

**Figure 1:**
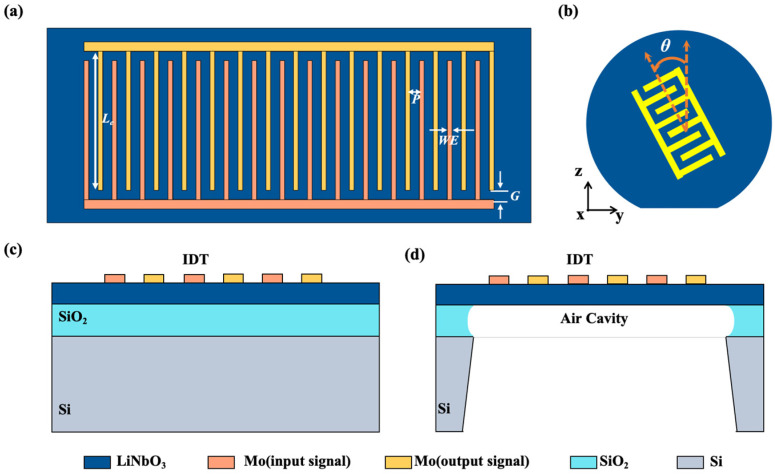
(**a**) Two-dimensional cross-sectional view of the IDT resonator. (**b**) Schematic diagram of the θ in the yz-plane. (**c**) The cross-sectional view of a resonator based on an X-cut LiNbO_3_/SiO_2_/Si substrate without a cavity. (**d**) The cross-sectional view of a resonator based on an X-cut LiNbO_3_ thin film with a cavity.

The vertical view of the resonator with an interdigital transducer electrode (IDT) in the xy-plane is shown in Figure 1a, where the main geometric parameters of the resonator are marked. *WE* and *L_e_* are the width and length of the electrode. The gap (*G*) is the distance between the electrode and the busbar. The pitch (*P*) is the center distance between adjacent electrodes. The angle at which the arrangement direction of the forks rotates counterclockwise along the y-axis is defined as θ. The resonator based on an X-cut LiNbO_3_/SiO_2_/Si substrate without a cavity, shown in Figure 1c, consists of a metal layer, a thin monocrystalline LiNbO_3_ layer, and a SiO_2_ layer supported by the Si substrates, while the resonator based on an X-cut LiNbO_3_ thin film with a cavity only consists of the metal layer and a thin-film piezoelectric layer, without SiO_2_ and Si as substrates. In addition, *h_Mo_*, *h_LN_*, and *h_SiO2_* represent the thickness of the metal layer, the LiNbO_3_ thin film, and the SiO_2_ layer, respectively.

The finite element simulation method (FEM) using COMSOL6.0 software is adopted in this article to simulate the dispersion characteristics of the resonators on an X-cut LiNbO_3_ thin film and the effects of *h_LN_* and θ on the resonant frequency. Periodic boundary conditions in the propagation direction (x-direction) and shear horizontal direction (y-direction) are applied.

**Figure 2:**
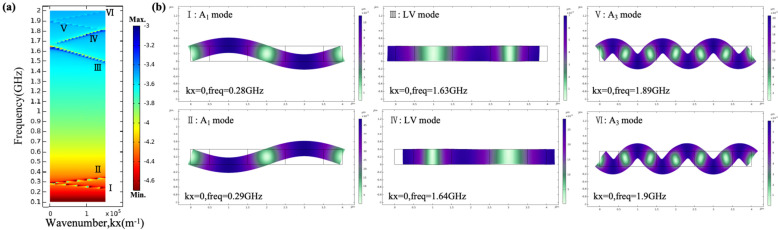
(**a**) The dispersive diagram for propagation on the x-axis for the real part of the propagation constant. (**b**) The total displacements of different mode shapes while kx = 0 m^−1^.

The dispersive diagram for propagation on the x-axis for the real part of the propagation constants (kx) is shown in Figure 2a. The dispersion curve presented in Figure 2a is different from that for Lamb waves, since the curve includes kx = 0 m^−1^ but also all multiples of pi/*P*. Figure 2b shows the total displacements of different mode shapes while kx = 0 m^−1^. Mode I and the mode II are the first-order anti-symmetric (A_1_) modes. Mode III and mode IV are the lateral vibration (LV) modes. The displacements of mode I have no shear displacement, which is different from the displacement of the main mode of the XBAR based on a Z-cut LiNbO_3_ thin film. Mode V and mode VI are the higher-order anti-symmetric modes, which are the third order antisymmetric mode (A_3_). One mode corresponds to two eigenfrequencies. A pair of eigenfrequencies of a mode represent the two edges of the bandgap. The displacements of this pair of eigenfrequencies are symmetric and asymmetric about the centerline, respectively. The appearance of the symmetric modes is due to constructive interference between two waves propagating in the opposite directions at one edge of the bandgap, causing resonance. The appearance of anti-symmetric modes is due to their destructive interference at the other edge of the bandgap, causing anti-resonance.

**Figure 3:**
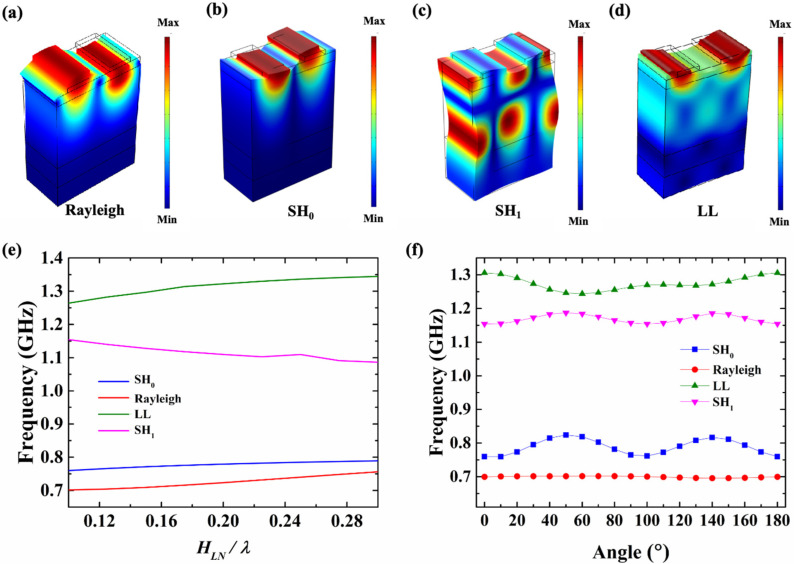
The mode shape of (**a**) Rayleigh SAW, (**b**) SH_0_-SAW, (**c**) SH_1_-SAW, and (**d**) LL-SAW. (**e**) The simulated frequencies of the four modes varies with the *H_LN_*/λ. (**f**) The simulated frequencies of the four modes varies with the θ.

In this paper, the modes of the resonator are analyzed firstly. Figure 3a–d show the four main modes in the SAW resonators based on an X-cut LiNbO_3_/SiO_2_/Si substrate, corresponding to the Rayleigh, the zero-order shear horizontal (SH_0_), the first-order shear horizontal (SH_1_), and the longitudinal leaky (LL), respectively. The FEM is adopted in this article to simulate the effects of *h_LN_* and θ on resonant frequency. Figure 3e shows the variation in the four modes’ frequencies with the variation in the *h_LN_*, while the *WE* is fixed at 1 μm and the *P* is 2 μm. From the graph, we can see that for the SH_0_-SAW resonators, the resonant frequencies of the SH_0_ mode and Rayleigh mode are always very close regardless of the *h_LN_*. This makes it important to suppress the Rayleigh mode for the SH_0_-SAW resonators. As the *h_LN_*/λ increases (the λ is the wavelength), the resonant frequencies of the SH_0_ mode show a slow upward trend but remain in the low-frequency range below 1 GHz. The resonant frequencies of the higher-order modes (the SH_1_ mode and the LL mode) are always above 1 GHz. Figure 3f reveals that the patterns of the simulated frequencies of the four modes vary with the θ of the resonators in the yz-plane, while the *h_LN_*/λ is fixed at 0.1. The horizontal setting of the IDTs in the yz-plane is defined as the initial value, with a θ of 0°. When the θ varies from 0° to 180°, the frequencies of the SH_0_ resonators exhibit periodic changes. The resonant frequency reaches the highest value at a θ of 50° and 140°. As the θ increases from 0° to 50°, the resonant frequency of the SH_0_ mode increases and then gradually decreases as the θ further increases to 90°. The SH_1_ mode and the SH_0_ mode have similar patterns of variation, while the frequency response of the Rayleigh waves is not sensitive to changes in the θ. For the LL mode, its maximum frequency response occurs when the θ is 0°.

**Figure 4:**
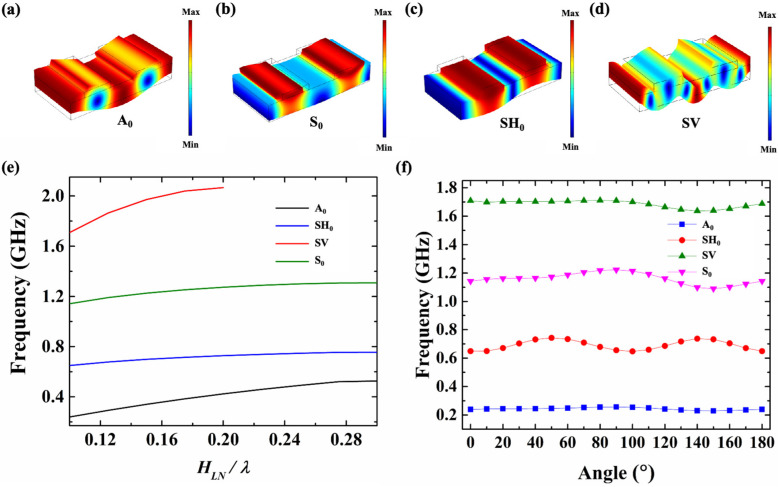
The mode shape of (**a**) the A_0_ mode, (**b**) the S_0_ mode, (**c**) the SH_0_ mode, and (**d**) the SV mode. (**e**) The simulated resonant frequencies of the four modes vary with the *h_LN_*/λ. (**f**) The simulated resonant frequencies of the four modes vary with the θ of the resonators in the yz-plane.

Figure 4a–d show the four main modes of resonators based on an X-cut LiNbO_3_ thin film with cavities, corresponding to the zero-order anti-symmetry (A_0_) mode, the zero-order symmetry (S_0_) mode, the SH_0_ mode, and the shear vertical (SV) mode, respectively. Figure 4e shows the resonant frequencies’ variation in the four modes with the different values of *h_LN_*/λ, while the *WE* is fixed at 1 μm and the *P* is 2 μm. For the resonators with the SH_0_ mode, as the *h_LN_* increases, the resonance frequencies’ variation in the SH_0_ mode is not significant, but the resonance frequencies’ variation in the A_0_ mode will gradually cause interference with the main resonance of the resonators. The frequencies’ variation in the S_0_ mode with the different values of *h_LN_*/λ are consistent with these of the SH_0_ mode, except that the frequencies are slightly higher. When *h_LN_*/λ increases to a certain level, the SV mode cannot be excited.

Figure 4f shows that the patterns of the simulated frequencies of the four modes vary with the θ, while the *h_LN_*/λ is fixed at 0.1. The frequencies of the SH_0_ mode exhibit periodicity as the θ changes from 0° to 180°, while the frequencies of the A_0_ mode have little effect on the change in the θ. The resonant frequency reached the highest value when the θ = 50° and θ = 140°. As the θ increases from 0° to 50°, the resonant frequency corresponding to the SH_0_ mode increases and then gradually decreases as the θ further increases to 90°. For the resonators with the S_0_ mode, the maximum value of frequency occurs when the θ = 90°. The SV mode can be excited at relatively high frequencies and maintains a certain difference from the main mode (S_0_) as the angle changes. Therefore, we can design the resonant frequency by rotating it without increasing the difficulty and complexity of the process, which provides new ideas for the design of filters.

### 2.2. Analysis of Rotation Angle

When the resonator rotates in the horizontal yz -plane, its performance changes accordingly, which is a noteworthy aspect of the resonator’s design on the X-cut LiNbO_3_ thin-film substrates. The FEM using COMSOL6.0 software is used for a simulation analysis and selected a 400 nm thick LiNbO_3_ thin film and a 200 nm thick Mo electrode. The thickness of the SiO_2_ is 3 μm, and there is a perfect matching layer under the Si substrate. In this part, the influence of the geometric structural parameters of the two types of resonators based on the X-cut LiNbO_3_ thin-film substrates on the resonant frequency and $K_{eff}^{2}$ are investigated. The $K_{eff}^{2}$ is calculated by the series resonant frequency (*f_s_*) and parallel resonant frequency (*f_p_*), as follows [33]:

(1) $$K_{eff}^{2}=\frac{\pi^{2}}{4}(f_{s}(f_{p}-f_{s}))/{f_{p}}^{2}$$

**Figure 5:**
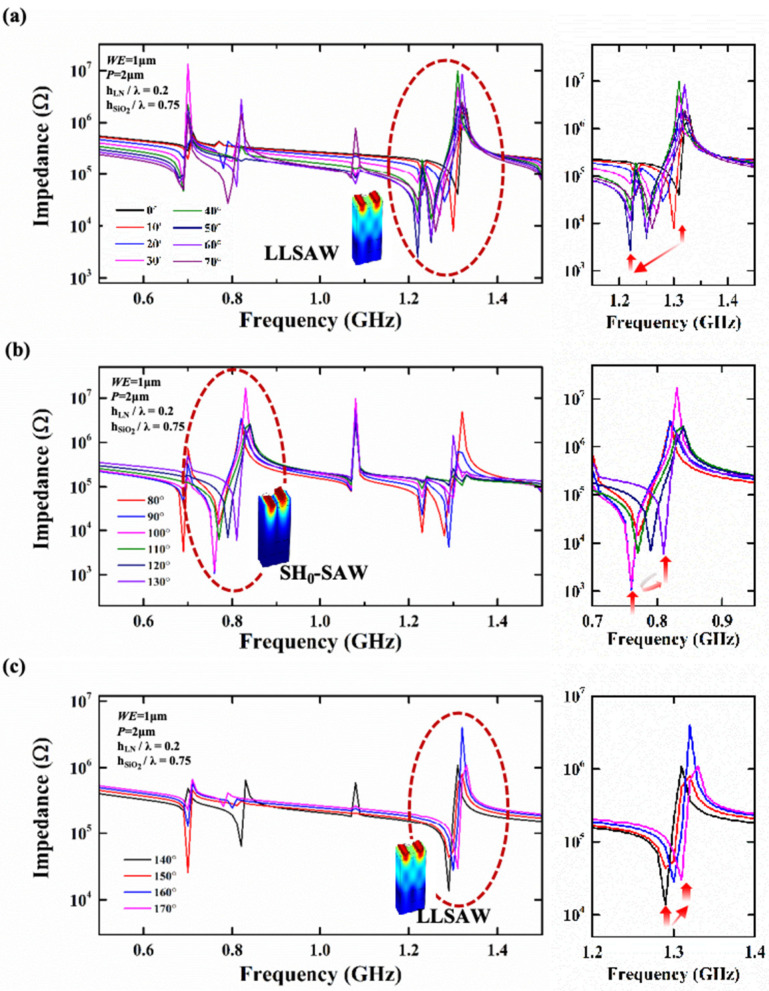
(**a**) The impedance curve of the corresponding resonator when the angle θ changes from 0° to 70°. (**b**) The impedance curve of the corresponding resonator when the angle θ changes from 80° to 130°. (**c**) The impedance curve of the corresponding resonator when the angle θ changes from 140° to 170°. The main resonance is located within the red dashed circle in the figure, and the red arrow marks the change in *f_s_*.

When the resonator rotates in the horizontal yz-plane, its performances change accordingly, which is a noteworthy aspect of the X-cut LiNbO_3_ thin-film substrates. For resonators based on an X-cut LiNbO_3_/SiO_2_/Si substrate, when the IDT electrodes are horizontally distributed along the y-axis (while θ = 0°), the main mode of the resonator is the LL mode. As shown in Figure 5a, when the θ gradually increases from 0° to 70° (with a fixed *WE* = 1 μm, P = 2 μm, *h_LN_*/λ = 0.2, and *h_SiO2_*/λ = 0.75), the main mode is unchanged but the *f_s_* gradually decreases, and the *f_p_* does not change much. At this time, the electromechanical conversion efficiency increases. When the θ increases to 40°, the parasitic mode next to the main mode has a greater impact on the resonator, which is worth noting in resonator design. It can be seen from Figure 5b that when the θ increases to 80°, the main mode changes to the SH_0_ mode. When the θ further increases from 80° to 130°, the main mode of the resonator remains in the SH_0_ mode, but the *f_s_* decreases firstly and then increases, while the *f_p_* remains unchanged. Therefore, according to the previous formula, the $K_{eff}^{2}$ increase firstly and then decrease. In Figure 5c, when the θ further increases to 140°, the main resonant mode returns to the LL mode. As the θ gradually increases from 140° to 170°, the *f_s_* of the resonator gradually increases and the $K_{eff}^{2}$ of the resonator does not change much.

**Figure 6:**
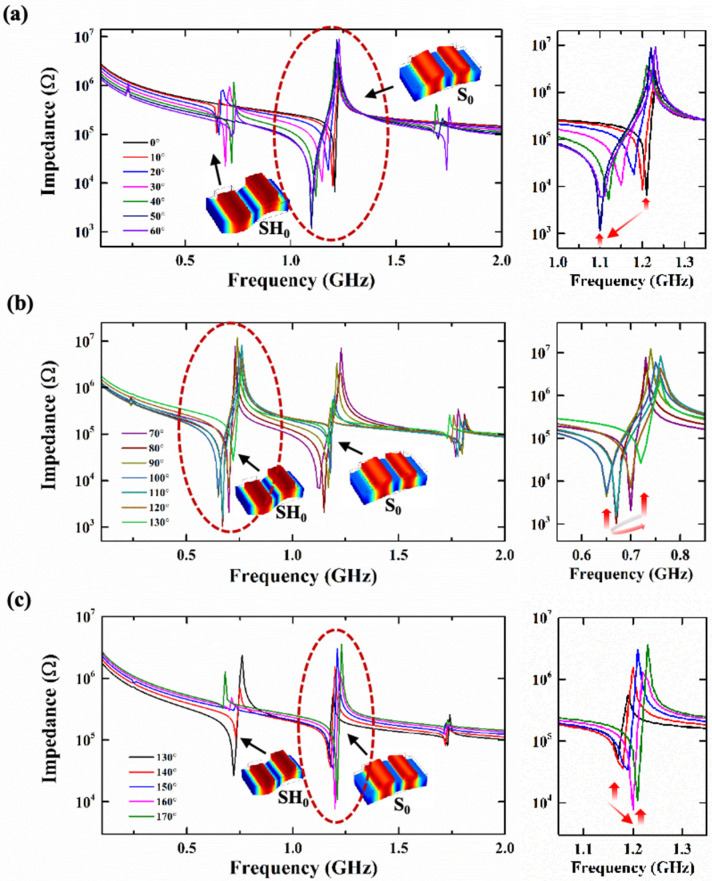
(**a**) The impedance curves of the corresponding resonators when the θ changes from 0° to 60°, (**b**) when the θ changes from 70° to 130°, and (**c**) when the θ changes from 130° to 170°. The main resonance is located within the red dashed circle in the figure, and the red arrow marks the change in *f_s_*.

For the resonators based on the X-cut LiNbO_3_ thin film with cavities, as the θ changes, its resonant mode is the SH_0_ mode or the S_0_ mode. When the θ is 0°, the main mode of the resonator is the S_0_ mode. As shown in Figure 6a, when the θ gradually increases from 0° to 60° (with a fixed *WE* = 1 μm, *P* = 2 μm, *h_LN_*/λ = 0.2, and *h_SiO2_*/λ = 0.75), the main mode remains unchanged, but the *f_s_* gradually decreases and the *f_p_* does not change much. At this time, the $K_{eff}^{2}$ increases. In Figure 6b, when the θ increases to 70°, the main mode of the resonator is the SH_0_ mode, and the operating frequency is less than 1 GHz. At this point, the resonator has a parasitic mode (S_0_) at higher frequencies. When the θ further increases from 70° to 130°, the main mode of the resonator remains in the SH_0_ mode, but the *f_s_* decreases first and then increases, and the $K_{eff}^{2}$ increase firstly and then decrease. As shown in Figure 6c, when the θ is 100°, the $K_{eff}^{2}$ reaches its maximum value. As the θ further increases, it gradually increases from 140° to 170°, and the main mode of the resonator switches back to the S_0_ mode. The *f_s_* of the resonators decrease as the θ increases.

**Figure 7:**
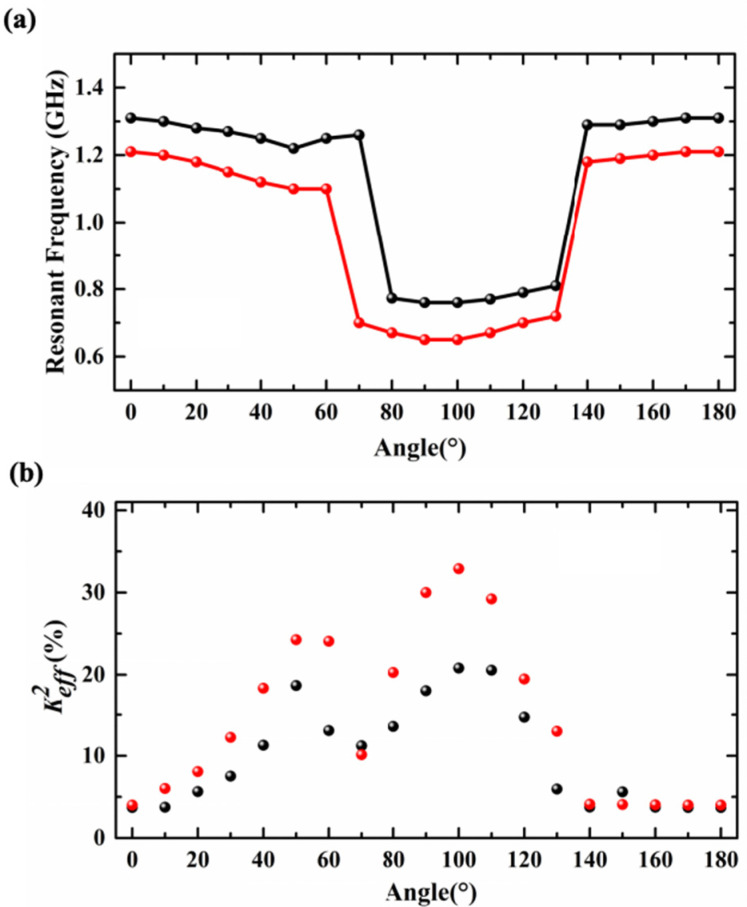
(**a**) The *f_s_* of the resonators with two types of substrates when the θ changes from 0° to 180°. The black line corresponds to the SAW resonators with an X-cut LiNbO_3_/SiO_2_/Si substrate, and the red line corresponds to the resonators with an X-cut LiNbO_3_ thin-film substrate. (**b**) The $K_{eff}^{2}$ of the resonator with two types of substrates when the θ changes from 0° to 180°. The black dots correspond to the SAW resonators with an X-cut LiNbO_3_/SiO_2_/Si substrate, and the red dots correspond to the resonators with an X-cut LiNbO_3_ thin-film substrate.

Figure 7a,b show the *f_s_* and $K_{eff}^{2}$ of the resonators based on an X-cut LiNbO_3_/SiO_2_/Si substrate and a LiNbO_3_ thin-film substrate when the θ changes from 0° to 180°; the black corresponds to the SAW resonators with an X-cut LiNbO_3_/SiO_2_/Si substrate and the red corresponds to the resonators with an X-cut LiNbO_3_ thin-film substrate. The corresponding specific data are presented in Table 1 and Table 2. For the SAW resonators with an X-cut LiNbO_3_/SiO_2_/Si substrate, when the θ is between 0° and 70°, the *f_s_* of the resonators is significantly higher than that when the θ is between 80° and 130°, which is maintained above 1 GHz. This is because within this range the main mode of the resonator is the LL mode. The $K_{eff}^{2}$ of the SAW resonators increase with the increase in the θ. When the θ is 50°, the sudden increase in $K_{eff}^{2}$ is due to the disturbance of the parasitic modes. When the θ is between 80° and 130°, due to the change of the main resonant mode to the SH_0_ mode, which has a low sound velocity, the *f_s_* decreases. The $K_{eff}^{2}$ increase firstly and then decrease as the θ increase. When the θ is 100°, the $K_{eff}^{2}$ reaches its maximum value, which is 20.81%. As the θ increases from 130° to 180°, the *f_s_* of the resonators is above 1 GHz, and as the θ increases, both *f_s_* and $K_{eff}^{2}$ of the resonators remain relatively constant. For the resonators with an X-cut LiNbO_3_ thin-film substrate, similar to the SAW resonators, when the θ is between 0° and 60°, the *f_s_* of the resonators are significantly higher than that when θ is between 70° and 130°. The *f_s_* of the resonators are above 1 GHz because the main mode of the resonator is the S_0_ mode. The $K_{eff}^{2}$ of the resonators increase with the increase in the θ. When the θ is between 70° and 130°, due to the change of the main mode to the SH_0_ mode, which has a low sound velocity, the *f_s_* decrease. The $K_{eff}^{2}$ increase firstly and then decrease as the θ increase. When the θ is 100°, the $K_{eff}^{2}$ reaches its maximum value, which is 32.9%. As the θ increase from 130° to 180°, the *f_s_* of the resonators are above 1 GHz. At this stage, the θ has little effect on the *f_s_* and the $K_{eff}^{2}$. The *f_s_* of the resonators with an X-cut LiNbO_3_ thin-film substrate are generally lower than that of the resonators with an X-cut LiNbO_3_/SiO_2_/Si substrate, but the $K_{eff}^{2}$ are larger than the $K_{eff}^{2}$ of the resonators with an X-cut LiNbO_3_/SiO_2_/Si substrate.

## 3. Fabrication and Characterization

**Figure 8:**
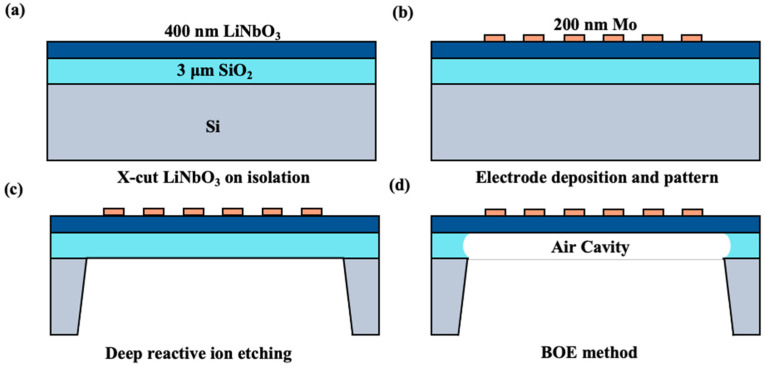
The process flow of the resonator: (**a**) the schematic diagram of the LNOI wafer; (**b**) pattern of the top electrode by the IBE method; (**c**) the DRIE method; (**d**) suspension of the resonator using the BOE method.

The fabrication process flow is illustrated in Figure 8a–d. The top electrode is a 200 nm-thick Mo layer to reduce the electrical loss of the electrodes, and the electrode was patterned using the ion beam etching (IBE) method. Later, the deep reactive ion etching (DRIE) method was used to suspend the resonators, and then we controlled the time precisely to remove the SiO_2_ by the buffered oxide etching (BOE) method.

**Figure 9:**
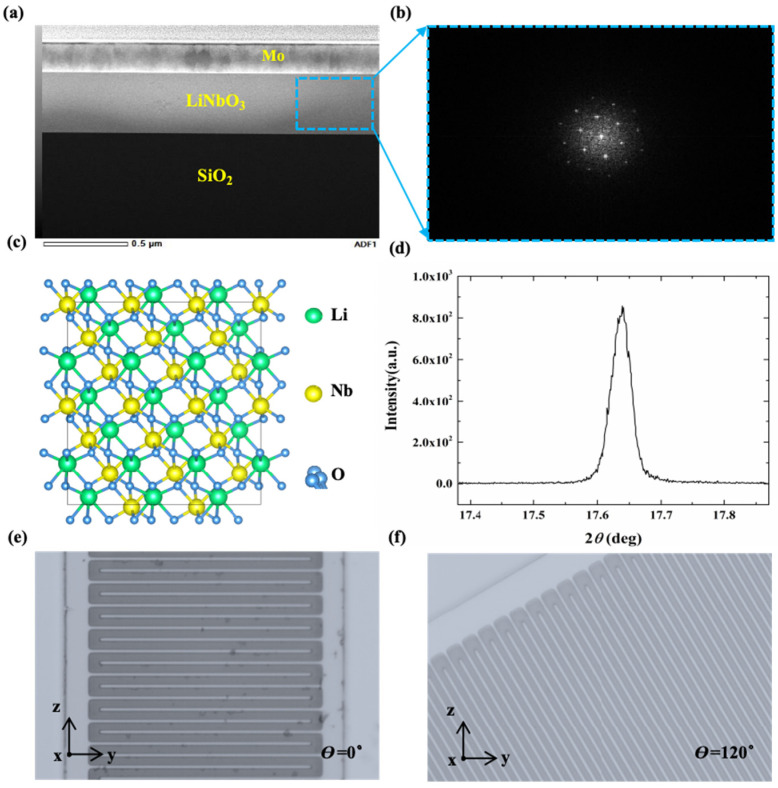
(**a**) Cross-sectional SEM images; (**b**) TEM image in LiNbO_3_; (**c**) schematic diagram of the unit cell of X-cut LiNbO_3_; (**d**) measured XRD spectrum of the LiNbO_3_ film; (**e**) optical image of a resonator with a θ of 0°; (**f**) optical image of a resonator with a θ of 120°.

The cross-sectional scanning electron microscope (SEM) images are shown in Figure 9a. The picture in Figure 9b is the transmission electron microscope (TEM) image in the X-cut LiNbO_3_. A schematic diagram of the unit cell of the X-cut LiNbO_3_ is shown in Figure 9c, and the measured X-ray diffraction (XRD) spectrum of the LiNbO_3_ film is shown in Figure 9d. The optical images of the devices of a LiNbO_3_/SiO_2_/Si membrane with IDTs having dimensions of pitch of 2 μm and electrode width of 1 μm are shown in Figure 9e,f. The devices were fabricated on a 400 nm-thick X-cut LiNbO_3_/SiO_2_/Si substrate. The three-layer structure of the LiNbO_3_ on isolation (LNOI) wafer is 400 nm-thick LiNbO_3_, 3 μm-thick SiO_2_, and 725 μm-thick Si, respectively.

### 3.1. Impedance Curve and MBVD Model Fitting

**Figure 10:**
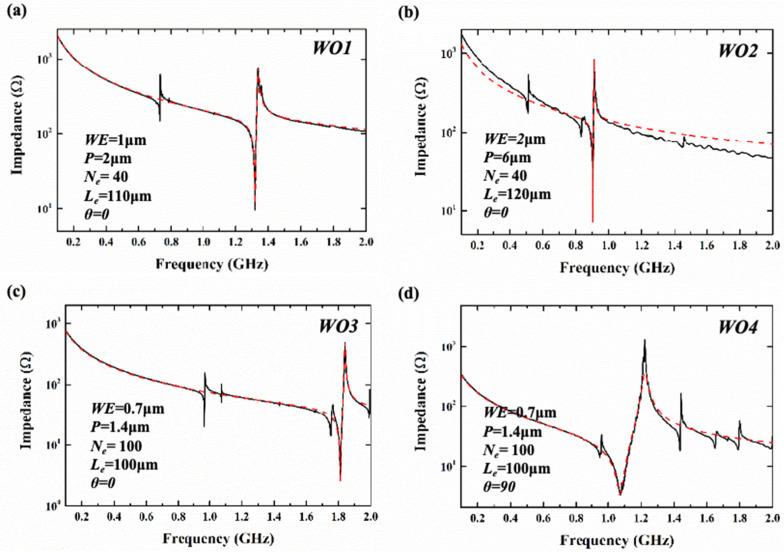
The measured impedance curves (the black line) and MVBD fitting curves (the red line) of (**a**) WO1, (**b**) WO2, (**c**) WO3, and (**d**) WO4.

**Figure 11:**
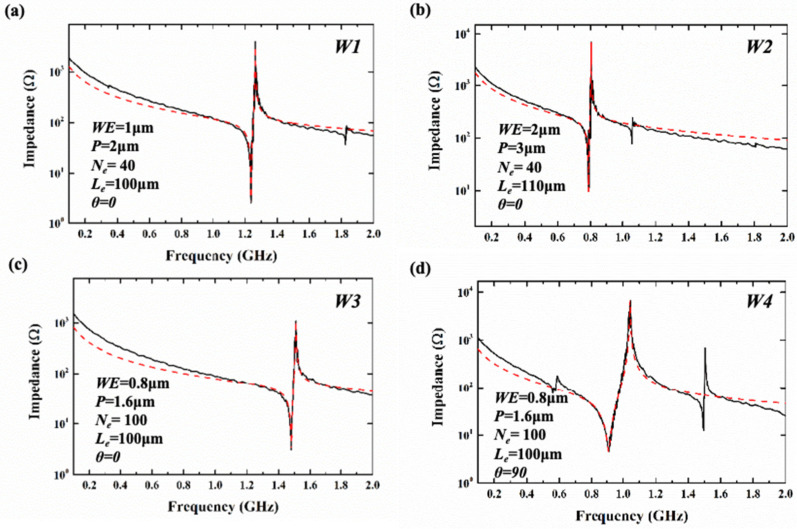
The measured impedance curves (the black line) and MVBD fitting curves (the red line) of (**a**) W1, (**b**) W2, (**c**) W3, and (**d**) W4.

With a ground–signal–ground (GSG) probe and an Agilent N5222B vector network analyzer platform, the S parameters (*S*_11_) of the resonators were measured and later converted to impedance via software. Figure 10 and Figure 11 show the corresponding measured impedance curves and Modified Butterworth–Van Dyke (MBVD) modal fitting curves of the resonators based on the two types of substrates. Finally, the $K_{eff}^{2}$, the static capacitance (*C*_0_), the maximum of Bode-*Q* (Bode-*Q*_max_), and the resistance parameters were extracted and are displayed in Table 3 and Table 4. The Bode-*Q* is evaluated using the following equation [29]:

(2) $$Q_{B\text{ode}}=\omega \;\cdot \;|S_{11}|\;\cdot \;\frac{GD(S_{11})}{1-|S11|}$$

where the *ω* represents the angular frequency, the *S*_11_ is the one port scattering parameter, and *GD*(*S*_11_) is the group delay derived from *S*_11_. The results of the measurement are consistent with the results of the simulation, and the introduction of the θ has a regulating effect on the *f_s_* and $K_{eff}^{2}$ of the resonators. From the test results, it can be seen that for resonators based on the two types of the substrates, the resonators with a θ of 0° at different frequencies have high-quality factors (*Q*), and the lower $K_{eff}^{2}$ of the resonators can be greatly improved by rotating the IDT. A LLSAW resonator obtained a high Bode-*Q_max_* of 1309 and a $K_{eff}^{2}$ of 1.62% at 904 MHz. The $K_{eff}^{2}$ of a SH_0_-SAW resonator with a θ of 90° is 26.6%, and the Bode-*Q*_max_ is 228. For resonators with cavities, the resonator obtained a Bode-*Q_max_* of 1237 and a $K_{eff}^{2}$ of 5.16% at 1.237 GHz. The $K_{eff}^{2}$ of the SH_0_-PAW resonator is 27.66% and the Bode-*Q*_max_ is 288.

### 3.2. Impact of Rotation Angle

**Figure 12:**
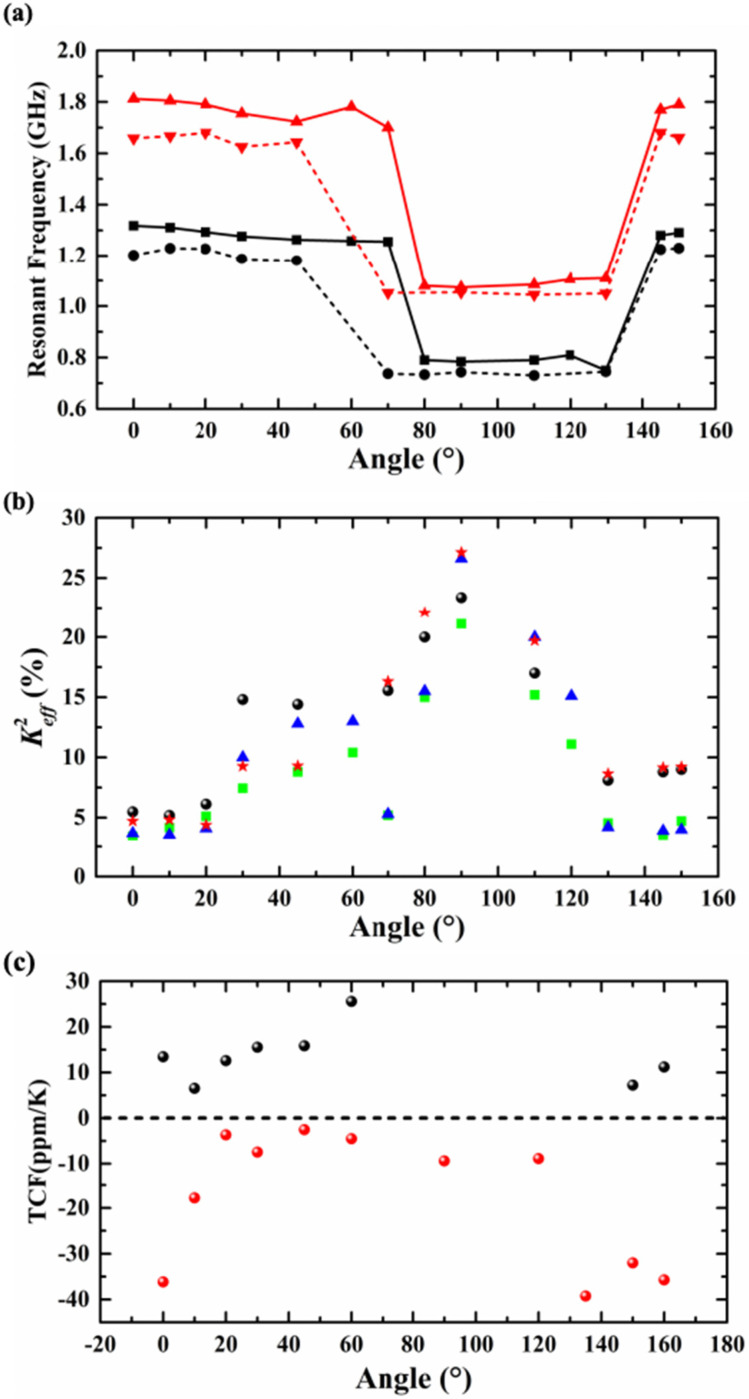
(**a**) The measured *f_s_* of the resonators based on an X-cut LiNbO_3_/SiO_2_/Si substrate and a LiNbO_3_ thin film when the θ changes from 0° to 180°. The black solid line corresponds to resonators with an X-cut LiNbO_3_/SiO_2_/Si substrate while *WE* = 1 μm and *P* = 2 μm, and the black dashed line represents the resonators with an X-cut LiNbO_3_/SiO_2_/Si substrate while *WE* = 0.7 μm and *P* = 1.4 μm. The red solid line corresponds to the resonators with an X-cut LiNbO_3_ thin-film substrate while *WE* = 1 μm and *P* = 2 μm, and the red dashed line represents the resonators with an X-cut LiNbO_3_ thin-film substrate while *WE* = 0.7 μm and *P* = 1.4 μm. (**b**) The $K_{eff}^{2}$ of the resonators with two types of substrates when the θ changes from 0° to 180°. The green square corresponds to the resonators with an X-cut LiNbO_3_/SiO_2_/Si substrate while *WE* = 1 μm and *P* = 2 μm, and the black dots represent the resonators with an X-cut LiNbO_3_/SiO_2_/Si substrate while *WE* = 0.7 μm and *P* = 1.4 μm. The blue triangle corresponds to the resonators with an X-cut LiNbO_3_ thin-film substrate while *WE* = 1 μm and *P* = 2 μm, and the red pentagram represents the resonators with an X-cut LiNbO_3_ thin-film substrate while *WE* = 0.7 μm and *P* = 1.4 μm. (**c**) The measured TCFs of the resonator with two types of substrates when the θ changes from 0° to 180°. The black dots correspond to the resonators with an X-cut LiNbO_3_/SiO_2_/Si substrate, and the red dots correspond to the resonators with an X-cut LiNbO_3_ thin-film substrate.

Figure 12a,b show the measured *f_s_* and $K_{eff}^{2}$ of the resonators based on an X-cut LiNbO_3_/SiO_2_/Si substrate and a LiNbO_3_ thin film when the θ changes from 0° to 180°. The results of the measurement are in good agreement with the results of the simulation. From Figure 12a, it can be seen that regardless of the change in *WE*, when the θ increases from 0° to 60°, the *f_s_* of the resonators based on an X-cut LiNbO_3_/SiO_2_/Si substrate and a LiNbO_3_ thin film change very little and remain stable above 1 GHz. The $K_{eff}^{2}$ increases with the increase in the θ (as can be seen from Figure 12b). As the θ continues to increase, the main modes of the resonators are changed. Meanwhile, the sound speed and the *f_s_* of the resonators decrease. The $K_{eff}^{2}$ reaches its maximum when θ is 90°, while for the resonators based on an X-cut LiNbO_3_/SiO_2_/Si substrate, the maximum of $K_{eff}^{2}$ is 26.6%, and for the resonators based on a LiNbO_3_ thin film, the maximum of $K_{eff}^{2}$ is 27.12% (both *WE* = 0.7 μm and *P* = 1.4 μm). The reason for the deviation between this point and the simulation value is that the resonators were damaged during fabrication. When the θ is 150°, the *f_s_* returns to 1.66 GHz while *WE* = 0.7 μm and *P* = 1.4 μm, and meanwhile, the $K_{eff}^{2}$ is 9.21%. The temperature coefficient of frequency (TCF) of the fabricated devices with two types of substrates were measured, as shown in Figure 11c. The first-order TCF of the series resonant frequency is defined as follows:

(3) $$TCF=\frac{1}{f_{s}}\frac{∆f_{s}}{∆T}$$

The TCFs of the resonators based on an X-cut LiNbO_3_/SiO_2_/Si substrate are positive, while the TCFs of the resonator based on an X-cut LiNbO_3_ thin film are negative. The TCFs of the resonators without cavities are compensated due to the SiO_2_ below the LiNbO_3_ layer, which has the opposite temperature coefficient of the LiNbO_3_ material. The TCFs of the resonators based on an X-cut LiNbO_3_/SiO_2_/Si substrate increases from 6.54 ppm/K to 25.58 ppm/K and minimize at 6.54 ppm/K when θ is 10°. For the resonators based on an X-cut LiNbO_3_ thin film, its TCFs increases from −39.25 ppm/K and maximums at −3.67 ppm/K when θ is 20°. The TCF is an important reference index for the subsequent application of resonators, so it needs to be carefully considered in resonator design.

## 4. Conclusions

In this study, the performances of the resonators based on an X-cut LiNbO_3_ thin film without and with cavities were investigated theoretically and experimentally. When the main modes of the resonators have high acoustic velocity, such as the LL mode (θ is 0°–70° and 140°–170°) and the S_0_ mode (θ is 0°–60° and 140°–170°), the *f_s_* of the resonators are increased. The $K_{eff}^{2}$ reaches its maximum at a θ of 100°. For the SAW resonators, the maximum of $K_{eff}^{2}$ is 20.81%, while for the LVRs, the maximum of $K_{eff}^{2}$ is 32.9%. The fabricated LLSAW (θ = 0°) and a SH_0_-SAW resonator (θ = 90°) present the $K_{eff}^{2}$ of 1.62% and 26.6% and the Bode-*Q_max_* of 1309 and 228, respectively. The fabricated S_0_ LVR (θ = 0°) and a SH_0_-PAW (θ = 90°) present the $K_{eff}^{2}$ of 4.82% and 27.66% and the Bode-*Q_max_* of 3289 and 289, respectively. In addition, we obtained a LVR with a low TCF of −3.67 ppm/K while the θ is 20° and a LLSAW with a low TCF of 6.54 ppm/K while the θ is 10°. This work provides potential strategies for multi-band and multi-bandwidth filters.

## Figures and Tables

**Table 1:** The *f_s_* and (b) $K_{eff}^{2}$ of the resonator without a cavity when the θ changes from 0° to 180°.

θ **(°) ^1^**	0	10	20	30	40	50	60	70	80
***f_s_* (GHz) ^2^**	1.31	1.3	1.28	1.27	1.25	1.22	1.25	1.26	0.773
$K_{eff}^{2}$ **(%) ^3^**	3.71	3.74	5.65	7.53	11.3	18.69	13.08	11.22	13.57
θ **(°) ^1^**	90	100	110	120	130	140	150	160	170
***f_s_ *(GHz) ^2^**	18.05	20.81	20.56	14.69	5.95	3.77	5.61	3.74	3.71
$K_{eff}^{2}$ **(%) ^3^**	30	32.9	29.22	19.48	12.99	4.11	4.08	4.04	4.01

^1^ θ is the rotation angle of the resonators; ^2^ *f_s_* is the series resonant frequency; ^3^ $K_{eff}^{2}$ is the effective electromechanical coupling coefficient of the resonators.

**Table 2:** The *f_s_* and (b) $K_{eff}^{2}$ of the resonator with a cavity when the θ changes from 0° to 180°.

θ **(°) ^1^**	0	10	20	30	40	50	60	70	80
***f_s_* (GHz) ^2^**	1.21	1.2	1.18	1.15	1.12	1.1	1.1	0.7	0.67
$K_{eff}^{2}$ **(%) ^3^**	4.01	6.02	8.09	12.24	18.35	24.27	24.07	10.14	20.28
θ **(°) ^1^**	90	100	110	120	130	140	150	160	170
***f_s_ *(GHz) ^2^**	0.65	0.65	0.67	0.7	0.72	1.18	1.19	1.2	1.21
$K_{eff}^{2}$ **(%) ^3^**	30	32.9	29.22	19.48	12.99	4.11	4.08	4.04	4.01

^1^ θ is the rotation angle of the resonators; ^2^ *f_s_* is the series resonant frequency; ^3^ $K_{eff}^{2}$ is the effective electromechanical coupling coefficient of the resonators.

**Table 3:** Performance of fabricated devices with LNOI substrate.

Resonator	WO1	WO2	WO3	WO4
Mode	LLSAW	LLSAW	LLSAW	SH_0_-SAW
*f_s_* (GHz)	1.319	0.904	1.812	1.074
$K_{eff}^{2}$ (%)	3.45	1.62	3.70	26.60
Bode-*Q_max_*	708	1309	705	228
*C_0_* (pF)	0.736	1.197	2.06	3.641
*R_0_* (Ω)	27	19.55	1.3	1.5
*R_S_* (Ω)	0.408	0.65	0.4	1.2
*R_M_* (Ω)	9.31	6.6	2.2	2

**Table 4:** Performance of fabricated devices with an X-cut LiNbO_3_ thin film.

Resonator	W1	W2	W3	W4
Mode	S_0_	S_0_	S_0_	SH_0_
*f_s_* (GHz)	1.237	0.787	1.48	0.91
$K_{eff}^{2}$ (%)	5.16	4.82	4.65	27.66
Bode-*Q_max_*	1237	803	740	288
*C_0_* (pF)	1.202	0.876	1.881	1.87
*R_0_* (Ω)	1.9	1.4	0.76	0.5
*R_S_* (Ω)	1.1	3.65	1	0.48
*R_M_* (Ω)	1.4	5.99	2.12	4.1

## Data Availability

The original contributions presented in the study are included in the article; further inquiries can be directed to the corresponding author.
